# Beyond Efficiency Scores: Explaining Health System Performance Using Two-Stage Bootstrap DEA and Machine Learning

**DOI:** 10.3390/healthcare14162536

**Published:** 2026-08-13

**Authors:** Kübra Çakır, Melis Almula Karadayı

**Affiliations:** 1 School of Natural Sciences and Engineering, Istanbul Medipol University, Beykoz 34810, Istanbul, Türkiye; makaradayi@medipol.edu.tr; 2 Healthcare Systems Engineering, Istanbul Medipol University, Beykoz 34810, Istanbul, Türkiye

**Keywords:** health system performance, data envelopment analysis, Simar–Wilson Bootstrap, machine learning, partial dependence analysis, health policy

## Abstract

**Background/Objectives:** Health systems involve numerous stakeholders interconnected through nonlinear relationships. While Data Envelopment Analysis (DEA) has been widely used to measure health system efficiency, conventional estimates may exhibit finite-sample bias. An important question, therefore, concerns how health system performance can be measured more reliably, and what factors explain cross-country differences in efficiency. This study introduces an integrated framework that combines Two-Stage Bootstrap DEA with machine learning to assess the performance of the health systems of 26 OECD countries using 2022 data. **Methods:** In the first step, technical efficiency scores are computed using an output-oriented constant returns to scale (CRS) DEA model. Subsequently, bias-corrected efficiency estimates are derived using the Bootstrap procedure proposed by Simar and Wilson. In the second step, truncated regression analysis and machine learning-based partial dependence analysis, the latter validated through leave-one-out cross-validation, are employed to investigate the determinants of efficiency. **Results:** The Bootstrap procedure reveals statistically significant differences from conventional DEA results, and bias-corrected results indicate that South Korea, Canada, and the United States achieve the highest efficiency levels. The findings show that tobacco use prevalence has a significantly negative association with health system efficiency and alcohol consumption exhibits a negative, threshold-type pattern, while GDP per capita and out-of-pocket health expenditure display more complex, nonlinear effects. Furthermore, the scenario analysis indicates that a 10% reduction in tobacco use yields the largest predicted single-intervention improvement, while combined interventions produce additional but sub-additive gains. **Conclusions:** The proposed framework presents a transparent and validated approach for assessing and explaining health system performance, generating findings relevant to the development of evidence-based health policy.

## 1. Introduction

Health systems are crucial to improve public health [1]. Well-functioning health systems ensure the achievement of good health with efficient use of available resources [1,2]. Moreover, such systems are crucial to responding to health emergencies and reducing the burden of diseases [3]. In the last two decades, there have been many epidemics and pandemics that have affected the health systems. From the beginning of the 2000s, approximately 7.5 million people have died in these outbreaks [4,5].

The COVID-19 pandemic has been a major health problem that affects many people around the world [6,7]. During the COVID-19 pandemic, global healthcare spending rose substantially, reaching $9.8 trillion in 2021, equivalent to 10.3% of global GDP [8]. Out-of-pocket spending, which had declined in 2020, rebounded in 2021 across high-, upper-middle-, and lower-middle-income countries, returning to pre-pandemic levels [8]. These health crises have clearly demonstrated that countries should allocate their resources more effectively to have an efficient health system. It is emphasized that there is a need for performance assessment to ensure the effectiveness and continuity of healthcare services [9].

The World Health Organization (WHO) defined health systems as including “all activities whose primary purpose is to promote, restore, or maintain health” [10,11,12]. This definition is used as a perspective throughout the study. Health systems are multi-dimensional and complex systems. Due to their complex nature, the performance evaluation of health systems involves more uncertainty than in other sectors. Evaluating the performance of these systems helps identify areas for improvement and develop health policies accordingly. Unexpected crises, limited resources, and rising healthcare demands have increased the importance of these evaluations. In particular, these evaluations not only lead to an increase in investments but also enable the distribution of resources and monitoring of the outcomes of the infrastructures. As a result, to evaluate the complex nature of health systems and assess their performance, there is a need for a multi-dimensional approach that can evaluate the relationships between multiple inputs and outputs. Data Envelopment Analysis (DEA) is one of the most common methods for assessing the performance of a health system. However, the existing literature exhibits several limitations. Firstly, most of the studies use conventional DEA models that do not pay attention to sample size and statistical uncertainties. Simar and Wilson [13] developed the Bootstrap approach, later extended to Two-Stage settings [14], which is a powerful method to address biased performance scores. Secondly, the number of studies that evaluate health system performance with Two-Stage Bootstrap DEA is limited. Moreover, there is a literature gap with respect to studies that integrate machine learning with DEA. In addition, research employing a Two-Stage Bootstrap DEA to evaluate the health system performance of OECD countries remains limited. In this study, the performance of health systems in 26 countries of the Organization for Economic Co-operation and Development (OECD) is evaluated using the Two-Stage Bootstrap DEA method. In the first stage, it uses a model with constant returns to scale (CRS) and an output-oriented approach. However, due to its deterministic nature, conventional DEA is sensitive to sampling fluctuations, which may introduce bias into the efficiency scores and limit their reliability. To solve this shortcoming, a Two-Stage Bootstrap DEA is used in this study. This approach increases the statistical reliability of technical efficiency scores. In the second stage, truncated regression is employed to identify the explanatory variables that affect efficiency scores. Afterwards, Partial Dependence Analysis (PDA) and scenario analysis are applied to interpret the predictions of the trained machine learning models. While alternative interpretability methods, such as SHAP and LIME, provide valuable observation-level explanations, PDA is selected because it offers a global interpretation of predictor effects that directly supports scenario-based policy analysis. For completeness, SHAP-based variable importance results are reported as Supplementary Materials and are consistent with the PDA findings. Besides improving score accuracy, this integrated approach offers a more comprehensive view of the factors underlying these outcomes.

The main contributions of this study are as follows:

- Two-Stage Bootstrap data envelopment analysis and machine learning methods are combined in an analytical framework. The national health system’s performance is assessed and explained by this framework.
- The Simar–Wilson Bootstrap procedure is used to obtain bias-corrected efficiency estimates for 26 OECD countries, demonstrating the degree to which conventional DEA models may overestimate health system efficiency.
- To investigate potentially nonlinear and heterogeneous effects of specific environmental and behavioral determinants on efficiency, the analysis uses machine learning-based partial dependence techniques.
- To evaluate the possible impacts of alternative and combined policy interventions on efficiency, a policy-oriented scenario analysis based on machine learning models is carried out, providing information pertinent to health policy decision-making.

The remainder of the paper is structured as follows. Section 2 reviews the literature on health system efficiency at both national and hospital levels, along with studies applying machine learning in this area. Section 3 outlines the methodology and describes the dataset used in the analysis. Section 4 presents and discusses the empirical findings. Finally, Section 5 concludes the paper, highlighting its main limitations and suggesting directions for future research.

## 2. Literature Review

Health system performance assessment has been examined at both macro and micro levels in the literature. While macro-level analyses evaluate the overall performance of health systems across countries, micro-level analyses focus on hospitals, health facilities, or clinical practices. In the literature, DEA has been applied to evaluate the performance of Decision Making Units (DMUs) across a wide range of sectors, including banking [15], ecological efficiency [16], education [17], hospital efficiency [16,18,19,20], and health systems [18,21,22,23,24]. Although most of the studies focus on European and OECD countries, several of them examined Middle Eastern and Asian countries [25,26,27].

### 2.1. Bootstrap DEA Studies in Health Systems

Over the last decade, the literature indicates that Bootstrap DEA has become a robust method for measuring performance in health systems and health service providers [28,29,30,31]. However, there are relatively limited studies that have specifically observed health systems’ efficiency with the Two-Stage Bootstrap DEA. Marschall and Flessa [32] evaluated the efficiency of primary healthcare facilities in Burkina Faso using an input-oriented and CRS model based on 2004 data. Inputs included personnel costs, facility space, equipment depreciation, and vaccination-related costs expenditures; outputs included outpatient visits, family planning service, general construction, and vaccination counts. In the second stage, both truncated and Tobit regression are used for 16 explanatory variables. The average efficieny score is 0.88, and truncated regression provided a better model fit. Finally, the finding shows that there is a strong association between scores of household welfare, cultural-ethnic structure, and geographic location. A high average scores with only limited model dimensionality also illustrate a recurrent pattern in this study. De et al. [33] evaluated the health system performance of 29 Indian states and 2 union territories using Two-Stage Bootstrap DEA. In the first stage, an input-oriented CCR model is employed to estimate bias-corrected efficiency scores. The model incorporated the health expenditure per capita and income per capita as inputs, while life expectancy at birth and infant mortality rate are selected as outputs. In the second stage, Tobit regression is employed to investigate how socioeconomic indicators influence the efficiency scores. The results indicate that the average health system score efficiency in India is approximately 60%. According to the regression results, population size, female education, and immunization coverage are found to be significant determinants of efficiency, whereas health expenditure and the number of physicians did not have a significant effect. Notably, the study employs Tobit rather than the truncated regression recommended by [14]. Kim and Kang [34] assessed the efficiency of health systems for 170 countries, using the input-oriented BCC model. The analysis of health expenditure, and expected years of schooling for females aged 15 and above are chosen as inputs; life expectancy at birth, and under-five mortality rate served as outputs. A single-staged Bootstrap DEA is used; therefore, there are no explanatory variables. The results show that the majority of the countries are inefficient. Bias-corrected scores are low among high-income countries, averaging 0.74, whereas middle-income countries have scores between 0.66 and 0.77. However, being single-stage limits its policy relevance; as such, this study cannot explain the sources of this measured inefficiency. Li and Dong [35] assessed the technical efficiency of 14 public hospitals in China using the output-oriented conventional DEA model, followed by a Bootstrap-DEA to obtain bias-corrected efficiency scores. The model incorporated the number of hospital staff and available beds as inputs, whereas outpatient visits and inpatient discharges are defined as outputs. The results indicated that while eight hospitals are fully efficient under the conventional DEA model, after Bootstrap DEA, the number of efficient hospitals decreases. The study highlights the importance of applying the Bootstrap to obtain more reliable efficiency scores. Cheng et al. [36] examined the efficiency of health systems in rural hospitals across China, using five-year data covering the 2009–2013 period. The analysis examined both time-varying performance and relative efficiency levels across hospitals. A combined methodological framework is employed, including both Bootstrap DEA and the Malmquist productivity index. In the analysis, health personnel, the number of hospital beds, and total health expenditure are used as input, while outpatient visits, inpatient discharges, and the number of births are outputs. With the Malmquist index, the change over time in productivity is analyzed. The findings showed that the average technical efficiency of township hospitals is 75%, indicating considerable potential for improving service delivery with existing resources. A regional differentiation is clear; the hospitals in eastern China have higher efficiency than the others. Consequently, the study concludes that while recent health reforms have had positive effects on rural hospitals, regional inequalities persist, and further efforts are required. See and Yen [37] focuses on social indicators, unlike the other conventional studies that focus on financial and structural indicators to evaluate health system performance. Particularly, social well-being and life satisfaction influences are used. A Two-Stage Bootstrap DEA method is used to evaluate 121 countries. Firstly, an output-oriented CCR model is employed, where health expenditure per capita, health workforce, hospital beds, and educational attainment are used as inputs. The outputs are healthy life expectancy and the inverse mortality index. In the second stage, to address bias, three explanatory variables are included: the happiness index, population density, and the share of health expenditure in GDP. The findings revealed a positive relation between the happiness index and efficiency scores, suggesting that social well-being has a meaningful impact on health system performance. In contrast, population density exhibited weaker effects across countries. It is concluded that a Two-Stage approach generates more reliable efficiency estimates, underscoring the importance of integrating social determinants into health systems efficiency assessments. Asghar et al. [38] assessed the efficiency of healthcare service delivery among member countries of the Organization of Islamic Cooperation (OIC). Using data from 2016, the study evaluated the performance of 49 OIC countries with a Bootstrap DEA approach. The model used the number of health sector employees and the patient-to-physician ratio as inputs, while the number of individuals receiving healthcare per 1000 population, the inverse of the infant mortality rate, and average life expectancy are selected as outputs. Firstly, an input-oriented conventional DEA model is used, followed by Bootstrap bias correction to obtain more accurate and robust efficiency estimates. Although Chad, Niger, and Sudan achieved considerably poorer health outcomes than Malaysia, Qatar, the United Arab Emirates, and Türkiye, they exhibited high efficiency levels relative to their limited inputs. Mainly, these differences are due to socioeconomic conditions, health infrastructure, and the distribution of healthcare personnel. Andrews [39] evaluated the technical efficiency of the 20 District Health Boards in New Zealand’s public funds over the 2015–2019 period. To estimate efficiency scores, the study used a combined approach that combined Bootstrap DEA and beta regression. Total health expenditure and non-medical expenditure are included as inputs, while outpatient attendance rate, emergency department presentation rate, patient satisfaction, and timely access to appointments are selected as outputs. The average efficiency level is approximately 82%. While rural boards had lower performance scores, higher efficiency is seen in urban and densely populated areas. The regression findings also showed that ethnic diversity and socioeconomic disadvantage had a major impact on efficiency. This study highlights the need for better accessibility in rural areas and a comprehensive understanding of health systems performance that takes into account both financial and service quality aspects. Trakakis et al. [40] assessed the efficiency of human resources in public primary healthcare centers in Greece, focusing on the utilization of staff across facilities. The study applied a Two-Stage Bootstrap for 155 public health centers for the year 2016. Administrative personnel, nurses, and physicians are selected as inputs, whereas outpatient visits, home care services, and emergency department visits served as outputs. An input-oriented model is used. The findings revealed that only 28% of the centers are efficient, with the average score estimated at 74%. This indicates that health centers could potentially improve resource use by about 26%. Facilities located in rural areas exhibited lower performance compared to those in urban areas. It is noted that mismatches in human resource allocation, with certain centers experiencing understaffing while others have an oversupply of personnel, potentially compromise the quality and accessibility of care. Consequently, the results underscore the importance of aligning the human resource planning with service demand and addressing rural–urban differences to improve the effectiveness of primary healthcare in Greece. Konca and Top [41] evaluated the health systems performance of 36 OECD countries over the 2000–2016 period. In the first stage, an input-oriented CCR model is employed, using per capita health expenditure, physicians per 1000 population, and hospital beds per 1000 population as inputs. Two undesirable outputs—inverse infant mortality rate and inverse mortality from infectious disease—are incorporated after appropriate transformation. In the second stage, instead of relying solely on conventional economic indicators, a comprehensive set of ten socioeconomic, educational, and risk-factor variables is used: GDP per capita, economic growth rate, income inequality, unemployment rate, a dummy variable for the 2008 financial crisis, alcohol consumption, obesity prevalence, tobacco use, dependence ratio, and tertiary education attainment. The findings indicated that higher income levels and greater educational levels had a positive effect on efficiency scores. In contrast, income inequality, obesity rate, the financial crisis variable, and alcohol and tobacco consumption are associated with reduced scores. Consequently, the study emphasizes that performance is influenced by behavioral and socioeconomic environments in addition to resource availability. It should be noted, however, that incorporating undesirable outputs through inverse transformation is methodologically debatable, since such transformations are not invariant and can materially affect the resulting efficiency scores [42]. Zulfakhar Zubir et al. [43] evaluated the technical efficiency of the Malaysian Ministry of Health in delivering healthcare services over 30 years. The study employed a Bootstrap DEA. Number of physicians and number of nurses, number of hospital beds, and total health expenditure are used as inputs, while outpatient visits, inpatient admissions, and utilization of maternal and infant health services are selected as outputs. Using an input-oriented model with annual data from 1988 to 2018, a Two-Stage Bootstrap is applied. The results revealed the efficiency scores fluctuating across three decades, with notable declines during the 1997 Asian financial crisis and 2008 global economic crisis. The average efficiency score, which is 86%, indicates that the Ministry has progressively improved its utilization of the health resources. The results show that the economic crises have immediate and measurable adverse effects on health system efficiency, but these impacts can be mitigated with investments.

Viewed together, these studies reveal several recurrent methodological tensions rather than a settled practice. Second-stage specifications vary widely—Tobit [33,41]; truncated regression [32,37], and beta regression [39]. Even though Simar and Wilson [14] provides a formal justification only for the truncated model. This heterogeneity reflects a broader debate: Simar and Wilson [14] caution that the validity of any second-stage regression rests on a separability condition that is rarely tested in applied work, while McDonald [44] argues that Tobit and least squares estimators often produce results similar to truncated regression in practice. Model dimensionality and orientation also differ substantially across studies, which limits the comparability of reported efficiency levels. Finally, roughly half of the reviewed studies stop at bias correction without examining determinants, and none move beyond linear second-stage models toward the nonlinear patterns that behavioral risk factors may generate.

### 2.2. DEA–Machine Learning Hybrid Studies in Health Systems

A growing body of work combines DEA with machine learning in the health sector, although mostly at the facility or program level rather than for national health systems. An et al. [45] developed a clustering method termed “k-DEA weighted classification,” in which the weights underlying conventional DEA efficiency scores replace the distance metric of the k-means algorithm, applied to data from an online health platform in China, the method grouped physicians with similar efficiency profiles into nine clusters with distinct behavioral and service characteristics. Chuang et al. [46] evaluated the operational efficiency of Taiwanese hospitals with an input-oriented DEA, re-predicted the resulting efficiency classes with artificial neural networks, and used decision trees to extract the rules separating efficient from inefficient hospitals, finding bed capacity, physician numbers, and medical device utilization to be the most influential factors. Cosgun et al. [47] assessed the COVID-19 vaccination performance of US states with an output-oriented CRS DEA followed by five machine learning algorithms for score prediction, among which gradient boosting variants and random forest performed best, and used variable importance analysis to identify the distributed dose rate and COVID-19 death rate as the most critical determinants. In a related study, Cosgun et al. [48] evaluated 693 US nursing homes with an input-oriented CRS model and predicted the resulting scores with neural networks, decision trees, random forest, and support vector machines, with random forest achieving the best predictive accuracy. Sun et al. [49] examined telehealth service efficiency across 3118 US counties during the pandemic, combining input-oriented DEA with six machine learning algorithms, and found limited English proficiency to be the strongest determinant of telehealth efficiency. Taken together, these hybrid studies demonstrate the value of coupling DEA with machine learning for prediction, classification, and variable importance analysis. However, they share three limitations relevant to the present study: they operate at the facility or program level rather than the national health system level; none of them apply Bootstrap bias correction to the DEA scores that the machine learning models subsequently learn from; and none extend the analysis to policy-oriented scenario evaluation. As summarized in Table 1, the two strands of literature reviewed above illustrate that several gaps remain.

### 2.3. Research Gap and Contribution

**Figure 1:**
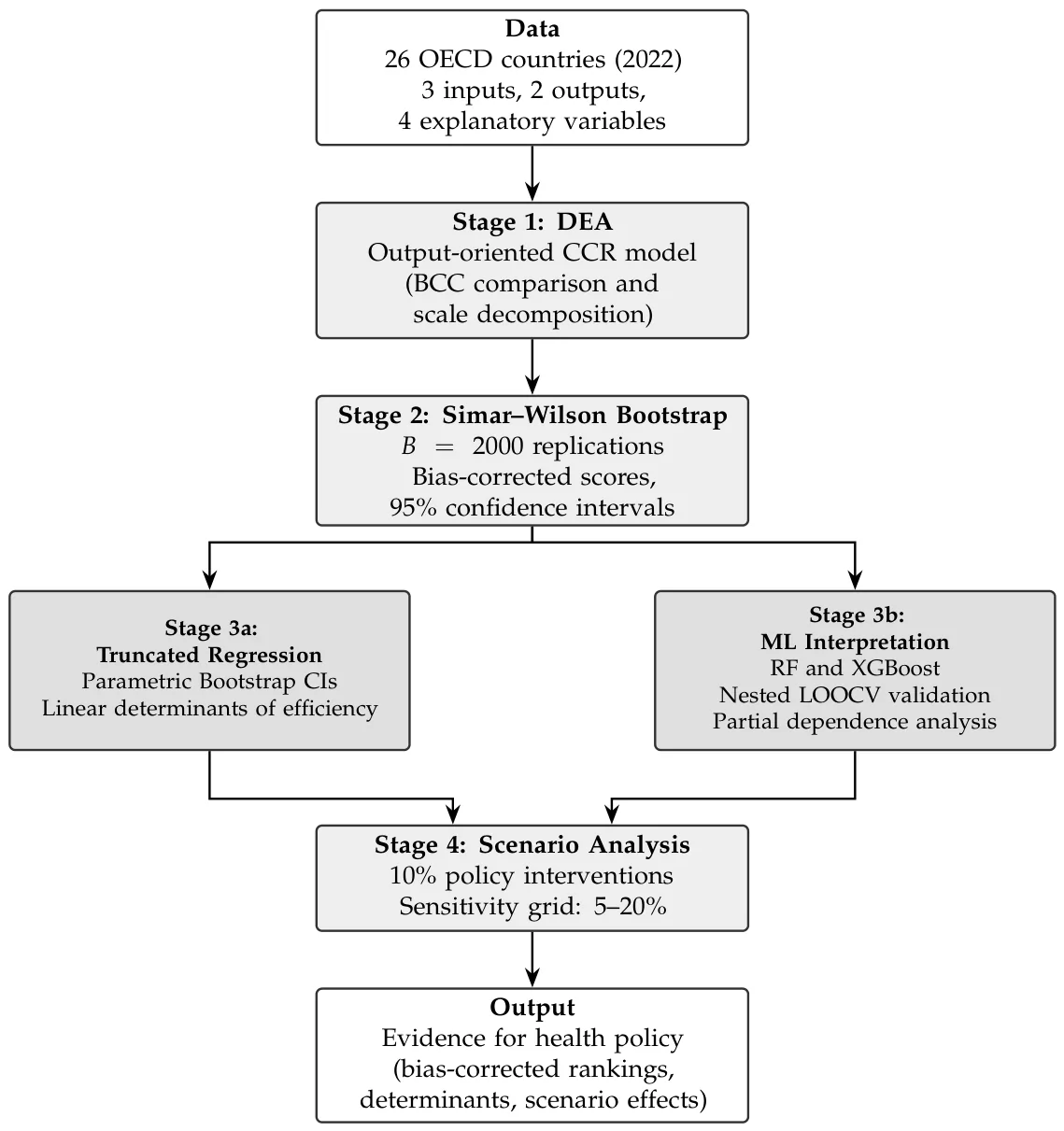
Overview of the proposed analytical framework.

Although there are many existing studies about the DEA to evaluate health system performance at both national and institutional levels, most existing studies rely on conventional DEA models and focus primarily on efficiency measurement rather than explanation. Moreover, empirical evidence employing bias-corrected DEA approaches in combination with machine learning techniques remains limited, particularly in the context of national health systems. Existing studies rarely address how efficiency estimates change after statistical bias correction, nor do they sufficiently explore the nonlinear and heterogeneous effects of environmental and behavioral factors on efficiency outcomes. In addition, the integration of efficiency measurement with predictive and scenario-based analyses is still underdeveloped in the health systems literature. Addressing these gaps, the present study combines a Two-Stage Bootstrap DEA framework with machine learning–based analytical tools to provide a more reliable assessment of health system efficiency, to examine key determinants of performance, and to explore the potential effects of alternative and combined policy interventions. This study contributes to the growing literature on health system efficiency by offering an integrated and policy-relevant analytical perspective. Figure 1 provides an overview of the proposed framework, which proceeds in four steps: conventional DEA estimation, Simar–Wilson bias correction, second-stage analysis through truncated regression and machine learning interpretation, and policy-oriented scenario analysis.

## 3. Methodology

DEA is first introduced by Farrell [50] in 1957, who conceptualized technical efficiency and proposed a framework based on linear programming. DEA is a non-parametric approach, which uses mathematical programming methods to estimate best-practice production frontiers and to evaluate the relative (technical or allocative) efficiency of DMUs [2]. Its suitability for health system performance assessment follows from the nature of the problem itself: health systems transform multiple resources into multiple outcomes, and the DEA evaluates this multi-input, multi-output process without requiring a pre-specified functional form or arbitrary indicator weights, in contrast to parametric alternatives such as Stochastic Frontier Analysis and composite index approaches. The extensive use of DEA in comparable OECD health system studies [41,51,52] also ensures the comparability of the present results with the existing evidence base. Over the past four decades, DEA has thus become one of the most widely used non-parametric efficiency measurement techniques [18,20,24]. DEA can be implemented through three different orientations:

- Input-oriented model, where the objective is to determine the extent to which inputs can be reduced while maintaining the same level of output for each DMU [53].
- Output-oriented model, which seeks to identify how much output can be expanded given the current level of inputs [53].
- Non-oriented model, which adopts a more comprehensive perspective by allowing simultaneous input reduction and output expansion that enables improvements in both directions at once [54,55].

Since health systems are complex systems in which input levels are often difficult to adjust and improving outputs is central to policy making, it is more appropriate to use an output-oriented model. Conventional DEA models do not account for sampling variation, which limits the statistical reliability of the estimated efficiency scores [13,56]. This issue becomes particularly pronounced in small sample settings, where the frontier is highly sensitive to the dimensionality of the model [13]. Moreover, because DEA scores are calculated relative to the best-performing units in the sample, they are inherently subject to bias and may overstate true efficiency levels.

### 3.1. CCR Model

The CCR model is initially proposed by Charnes et al. [57], and the model is named after the authors’ initials. It assumes that production has constant returns to scale (CRS), meaning any change in the input will result in a proportionate change in the output. Essentially, the CCR model evaluates all DMUs relative to a common production frontier, regardless of their size. The efficiency score reflects overall technical efficiency, encompassing both pure technical efficiency and efficiency at scale. This allows for the simultaneous evaluation of small- and large-scale units within a single, unified productivity framework.

In this study, an output-oriented CCR model is formulated as follows:

(1) $$\max_{\varphi ,\lambda }\;\varphi$$

(2) $$\mathrm{subject}\;\mathrm{to}\;Y\lambda \geq \varphi y_{0},$$

(3) $$X\lambda \leq x_{0},$$

(4) λ≥0,

where $X\in R^{m\times J}$ and $Y\in R^{t\times J}$ denote the input and output matrices, respectively; $x_{0}\in R^{m}$ and $y_{0}\in R^{t}$ represent the input and output vectors of the evaluated decision-making unit (DMU); $\lambda \in R^{J}$ is the intensity vector; and φ denotes the output expansion factor under constant returns to scale (CRS). This calculations are completed for the 26 countries, and technical efficiency is then reported as $1/\varphi^{*}$. A score of one identifies countries on the estimated frontier. The scores obtained from Equations (1)–(4) constitute the Stage 1 results reported in Section 4 and serve as the input for the Bootstrap procedure described in Section 3.2.

### 3.2. Bootstrap Model

Bootstrap is initially used in statistics by Efron [58] as a resampling technique for estimating the sampling distribution of estimators. This method aims to improve the distribution of estimates by repeatedly resampling the data. Because of its flexibility, Bootstrap methods have been widely adopted across various fields. Due to their deterministic nature and reliance on a single observed sample, conventional DEA estimates are sensitive to sampling variability and measurement error, which limits the statistical reliability of efficiency scores [14,19].

These issues are common in heterogeneous sectors such as healthcare systems, where efficiency comparisons are often conducted across decision-making units operating at different scales [16]. Moreover, since DEA efficiency scores are relative measures constructed with respect to the best-performing units, they may exhibit upward bias, especially in small samples [14]. Bootstrap DEA mitigates these limitations by generating samples through repeated resampling of the original dataset and re-estimating efficiency scores for each resample. The resulting empirical distribution of efficiency estimates is then used to obtain bias-corrected efficiency scores and confidence intervals, leading to more robust and statistically reliable performance assessments [59]. Bootstrap DEA allows for improved statistical inference compared to conventional DEA models with Monte Carlo simulation. Beyond bias correction, an important advantage of Bootstrap DEA is that it also has the ability to support statistical inference. The procedure improves the statistical reliability of efficiency comparisons across DMUs [60] and permits the development of confidence intervals by sample distribution.

### 3.3. Two-Stage Bootstrap DEA Model and Truncated Regression

Conventional DEA models’ efficiency scores are estimated deterministically and therefore do not account for measurement error or sampling uncertainty, which may lead to biased efficiency estimates. To address this limitation, Simar and Wilson [13] developed a Bootstrap procedure for nonparametric frontier estimators, and Simar and Wilson [14] extended this framework to two-stage settings, enabling bias correction and statistical inference regarding the efficiency determinants.

In the first stage, efficiency scores are obtained for each DMU using the conventional DEA model. These scores are calculated from a single observed sample; hence, they can be affected by data noise and sampling variation. The Bootstrap procedure mitigates this problem by repeatedly resampling from the original dataset and re-estimating efficiency scores for each Bootstrap sample, thereby generating an empirical distribution of efficiency estimates. The bias correction is performed on the Farrell output-expansion scores (${\hat{\varphi }}_{i}\geq 1$), on which the smoothed homogeneous Bootstrap of Simar and Wilson [13] is defined (B=2000):

(5) $${\hat{\varphi }}_{i}^{\;BC}=2{\hat{\varphi }}_{i}-\frac{1}{B}\sum_{b=1}^{B}{\hat{\varphi }}_{i}^{\;*b},$$

where ${\hat{\varphi }}_{i}$ denotes the original DEA estimate, ${\hat{\varphi }}_{i}^{\;*b}$ represents the estimate obtained from the *b*-th Bootstrap sample, and *B* is the total number of Bootstrap replications. The bias-corrected scores are then converted to the technical-efficiency scale, ${\hat{\theta }}_{i}^{\;BC}=1/{\hat{\varphi }}_{i}^{\;BC}\in \left(0,1\right]$, on which all results are reported; the bounds of the confidence intervals are inverted accordingly. The resulting bias-corrected scores provide more reliable estimates of underlying efficiency levels by filtering out sampling noise.

In the second stage, the bias-corrected efficiency scores obtained from Equation (5) are related to a set of environmental, structural, or socio-economic variables that may influence efficiency. Because the technical efficiency scores are bounded above by one, the use of ordinary least squares regression is inappropriate. Following the logic of Simar and Wilson [14], a truncated regression model with right truncation at unity is employed:

(6) $${\hat{\theta }}_{i}^{\;BC}=\beta_{0}+Z_{i}^{⊤}\beta +\varepsilon_{i},\;\varepsilon_{i}∼N\left(0,\sigma^{2}\right),\;{\hat{\theta }}_{i}^{\;BC}\leq 1,$$

where $Z_{i}$ is a vector of explanatory variables capturing external or contextual factors affecting efficiency. Inference on the regression coefficients is based on a parametric Bootstrap with 2000 replications, corresponding to the final steps of Algorithm 2 in Simar and Wilson [14]: truncated pseudo-observations are repeatedly generated from the estimated model, the regression is re-estimated on each sample, and percentile confidence intervals are constructed from the resulting empirical distributions. By jointly addressing bias correction and second-stage inference, the Two-Stage Bootstrap DEA framework allows for statistically reliable efficiency estimation while facilitating inference on the factors associated with performance differences across DMUs. These characteristics have led to its widespread use in performance studies, especially in multidimensional contexts such as health systems.

### 3.4. Data and Indicator Selection

To obtain a dataset that is homogeneous in terms of structural features, data availability, and international comparability, OECD countries are chosen as the DMUs. Although the OECD is composed of 38 member states, only 26 are included in the analysis because of limitations related to data availability and completeness. Data are sourced from the WHO and Our World in Data. The variables employed in this study are determined through a comprehensive review of the literature on health system performance assessment and are grouped into three categories: inputs, outputs, and explanatory variables. In this study, there are three input variables, two output variables, and four explanatory variables. The final selection of variables is shaped not only by earlier empirical research on health system performance but also by the availability and completeness of data across the 26 OECD countries, thereby ensuring methodological alignment with existing studies and allowing cross-country comparability.

To maintain adequate discriminatory power, the DEA model is intentionally limited to three inputs and two outputs. With 26 DMUs, the sample satisfies the common adequacy rule that the number of DMUs should be at least three times the sum of inputs and outputs (26>3×(3+2)=15) [54], but adding further dimensions would quickly erode this margin. Increasing the number of variables in a relatively small sample reduces the discriminatory power of DEA by increasing the proportion of units identified as efficient [13].

Therefore, the selected variables, shown in Table 1, represent the core input and output measures most consistently employed in cross-country health system efficiency studies, ensuring comparability with previous research while preserving the model’s ability to discriminate between countries. Among the output variables, infant mortality is incorporated as a survival rate rather than as an undesirable output. The transformation is a linear complement (1−IMR/1000), which is monotonic, preserves the scale of the original indicator, and yields a directly interpretable output (the proportion of infants surviving their first year). This differs from the multiplicative inversions (1/x) sometimes used in the literature, to which the non-invariance critique primarily applies [42]. Table 2 summarizes the variables used in the DEA framework, including their operational definitions, data sources, publication years, and representative reference studies, while Table 3 presents the descriptive statistics of the variables included in the study.

### 3.5. Model Selection

In this study, both the CCR model, which assumes constant returns to scale (CRS), and the BCC model, which assumes variable returns to scale (VRS), are estimated. Estimating both specifications makes it possible to decompose overall technical efficiency into pure technical and scale components and to assess which specification provides adequate discriminatory power for cross-country comparison, a known concern when a flexible VRS frontier is applied to a small sample [13]. Based on this comparison, the output-oriented CCR model, presented in Section 4, is retained as the main specification, while the BCC results are reported for comparison and for the decomposition of scale efficiency.

An output-oriented approach is adopted to reflect the policy-relevant objective of improving health outcomes rather than reducing inputs. Given constraints related to infrastructure, input reduction is often infeasible, whereas improving health outcomes with given inputs is more realistic and policy-consistent. The resulting efficiency scores represent countries’ technical efficiency levels. However, due to the deterministic nature of conventional DEA, these efficiency estimates may be subject to bias. To address this limitation, Simar-Wilson Bootstrap procedure is applied to conventional scores, completing the first stage of the analysis with bias-corrected efficiency estimates (Steps 1–2 in Figure 1). In the second stage, a truncated regression model is estimated to examine the effects of the explanatory variables on the bias-corrected efficiency scores (Step 3a).

### 3.6. Machine Learning-Based Models

Two tree-based ensemble methods are selected: Random Forest, which reduces variance through bagging, and XGBoost, which reduces bias through sequential boosting with explicit regularization. Using two ensembles with different learning paradigms allows the agreement between them to serve as a robustness check for the interpretation results. Tree-based methods are preferred over neural networks because they perform reliably on small tabular datasets, require fewer observations, and support partial dependence analysis directly. Gradient boosting is represented by XGBoost with its regularized implementation, which is better-suited to small samples than unregularized boosting.

#### 3.6.1. Random Forest Algorithm

One of the most popular ensemble techniques in statistical learning is the Random Forest (RF) algorithm, which is originally introduced by Ho [84] and later formalized by Breiman [85]. RF is a decision tree-based machine learning approach that is essentially a single-tree model by adding randomness to the tree construction process [85]. RF an ensembel machine learning method that extends decision-tree modeling by introducing randomness into both sample selection and tree construction [49,86] rather than choosing the best split from the entire set of predictors. This randomization increases ensemble diversity while lowering the correlation between individual trees. Instead of building a single tree, the RF algorithm uses Bootstrap samples of the original dataset to create a large number of unpruned decision trees, then aggregates their predictions to produce the final output. This aggregation relies on the bagging principle, which reduces model variance and improves predictive accuracy [87]. As a result, when compared to independent decision tree models, RF has a significantly lower risk of overfitting and a stronger capacity for generalization. Formally, the Random Forest prediction function can be expressed as:

(7) $$f_{\mathrm{RF}}\left(x\right)=\frac{1}{B}\sum_{b=1}^{B}T_{b}\left(x\right),$$

where *B* denotes the total number of trees in the ensemble, and $T_{b}\left(x\right)$ represents the prediction obtained from the *b*-th decision tree.

In this study, machine learning techniques are chosen to identify the factors influencing the bias-corrected efficiency scores obtained from the Bootstrap DEA stage. RF is selected due to its robustness against overfitting, its capacity to capture nonlinear interactions, and its availability of variable importance measures, which enhance interpretability. System-level and contextual indicators are employed as explanatory variables, and the dependent variable is the bias-corrected efficiency scores. The scikit-learn library (version 1.6.1) is used to implement the RF model in Python (version 3.12.13). An ensemble of 200 decision trees with a minimum node size of two observations and a fixed random seed of 42 is built to ensure reproducibility stable model performance and minimize variability.

#### 3.6.2. Extreme Gradient Boosting Algorithm

The Extreme Gradient Boosting (XGBoost) algorithm, introduced by Chen and Guestrin [88], is based on the gradient boosting framework and builds a strong predictive model by sequentially training several weak learners. In recent years, XGBoost has emerged as one of the most popular machine learning algorithms because of its high predictive accuracy, computational efficiency, and adaptable model structure [47,89]. XGBoost operates a sequential learning mechanism that is optimized to reduce the ensemble’s prediction errors. With this explicit regularization to control the risk of overfitting, the process enables the model to gradually improve its predictive performance [88,90]. The basic prediction function of XGBoost is given by:

(8) $${\hat{y}}_{i}=\sum_{k=1}^{K}f_{k}\left(x_{i}\right),\;f_{k}\in F,$$

where *K* denotes the total number of trees, and $f_{k}$ represents an individual regression tree belonging to the functional space F. The model objective function is defined as:

(9) $$\mathrm{Obj}=\sum_{i=1}^{n}l\left(y_{i},{\hat{y}}_{i}\right)+\sum_{k=1}^{K}\Omega \left(f_{k}\right),$$

where l(·) denotes the loss function measuring prediction error, and $\Omega (f_{k})$ is a regularization term that penalizes model complexity.

The main difference between RF and XGBoost lies in the structure of the decision tree construction. In RF, the decision trees are built independently of each other on Bootstrap samples and aggregated by averaging, whereas XGBoost constructs trees sequentially, with each new tree fitted to correct the errors of the previously created trees [90].

#### 3.6.3. Model Validation

Because the sample contains 26 observations, model validation is designed to avoid any information leakage between tuning and evaluation. Predictive performance is assessed with leave-one-out cross-validation (LOOCV): each country is predicted by a model trained on the remaining 25 countries. Hyperparameters are selected with nested cross-validation, using an inner 3-fold loop inside each LOOCV iteration, so that no observation is used to tune the model that predicts it. The final hyperparameters are then selected on the full dataset with the same procedure. As a stability check, repeated 5-fold cross-validation (50 repetitions) is also performed. Performance is reported with $R^{2}$, RMSE, and MAE in Section 4.

### 3.7. Partial Dependence Analysis

Partial dependence analysis (PDA) is originally proposed by Friedman [91] and later extended by Goldstein et al. [92]. Formally, the one-dimensional partial dependence function for a given explanatory variable $x_{S}$ is defined as:

(10) $${\hat{f}}_{\mathrm{PDP}}\left(x_{S}\right)=\frac{1}{n}\sum_{i=1}^{n}\hat{f}\left(x_{S},x_{C}^{\left(i\right)}\right),$$

where $x_{S}$ denotes the variable of interest, $x_{C}^{\left(i\right)}$ represents the values of all remaining variables for observation *i*, $\hat{f}\left(\cdot \right)$ is the prediction function of trained model, and *n* is the total number of observations. In this study, Equation 10 is evaluted separately for the trained Random Forest and XGBoost models, and the resulting curves are displayed jointly for each explanatory variable.

This formulation computes the average model prediction at a given value of $x_{S}$, thereby capturing the marginal effect of the selected variable on the predicted efficiency scores. PDA is conducted to improve the interpretability of the models’ structure. PDA plots show how efficiency scores vary across various ranges of the explanatory variables while controlling for the effects of all other variables [92]. PDA is utilized to examine the marginal associations between the explanatory variables and the bias-corrected efficiency scores derived from the Bootstrap DEA procedure. Subsequently, partial dependence plots are constructed for each explanatory variable using both ensemble models. By averaging over the joint distribution of the remaining variables, PDA provides a clear representation of nonlinear patterns and potential interaction effects that may not be fully captured by conventional regression-based approaches [93]. By isolating the effect of a single variable, the method facilitates the identification of value ranges in which health system performance is more sensitive to changes in specific contextual or system-level factors. To guard against over-interpretation in a small sample, a partial dependence pattern is interpreted only when two criteria are jointly satisfied: (i) the Random Forest and XGBoost curves agree on the direction and approximate location of the pattern, and (ii) the corresponding region of the predictor’s range contains a sufficient number of observations, as indicated by the rug marks in each plot. Segments failing either criterion are reported but not interpreted. The visualization and implementation procedures followed the methodological guidelines outlined by Greenwell [94], and the resulting plots are discussed in detail in the subsequent section.

### 3.8. Scenario Analysis with Machine Learning

Two ensemble machine learning algorithms, RF and XGBoost are used to implement the scenario analysis. Both of these algorithms are based on ensembles of decision trees; however, they differ fundamentally in their model-building strategies. The joint application of these two algorithms facilitated a more rigorous evaluation of scenario-based predictions by leveraging complementary ensemble learning paradigms. Both models are used with the hyperparameter configurations selected through the nested cross-validation procedure described in Section 3.6.3. The bias-corrected efficiency scores generated in the first stage (Steps 1–2) are used as the dependent variable, whereas the conventional DEA efficiency scores, together with the contextual explanatory variables, are included as predictors.

For each scenario, selected explanatory variables are systematically changed according to the intervention levels, and the models are re-estimated using the modified inputs. This procedure showed how the changes in policies affect the predicted efficiency scores. The 10% intervention level is selected as the primary magnitude because it corresponds to the scale of internationally agreed policy targets for the behavioral risk factors examined here, notably the WHO Global Action Plan target of a 10% relative reduction in the harmful use of alcohol [95]. To ensure that the conclusions do not depend on this choice, the analysis is additionally repeated at 5%, 15%, and 20% intervention levels, and the results are reported in Section 4. By integrating the statistically grounded results of the Bootstrap DEA with the predictive capabilities of machine learning, the analysis extends beyond a purely descriptive efficiency assessment toward a more prospective evaluation framework. The joint application of RF and XGBoost permits a quantitative assessment of potential efficiency gains or losses under alternative policy configurations and provides an empirically based foundation for scenario-specific interpretations of the potential evolution of health system performance under different intervention strategies. The policy scenarios examined in the machine learning-based analysis are summarized in Table 4.

## 4. Results and Discussion

This section presents the empirical results in four steps. First, the conventional DEA scores are reported together with the CCR-BCC comparison. Second, the bias-corrected estimates from the Bootstrap procedure are analyzed. Third, the truncated regression results are presented. Finally, the machine learning-based analyses, model validation, partial dependence, and policy scenarios are discussed. Table 5 summarizes the models employed throughout the analysis.

Using the conventional DEA framework, health system efficiency scores are estimated for the 26 OECD countries based on an output-oriented CCR model. Table 6 reports the CCR and BCC scores together with the implied scale efficiency. Under the CCR specification, scores range from 0.402 to 1.000, with a sample average of 0.688. A score of one represents full efficiency, while values below one suggest relative inefficiency [52].

Only two countries, Mexico and Türkiye, obtain a score of one and define the conventional frontier. South Korea (0.917), the United States (0.909), and Canada (0.901) follow closely, whereas Italy (0.518), Germany (0.514), Austria (0.442), and Greece (0.402) occupy the lower end. By comparison, under the VRS assumption, 21 of the 26 countries lie on the frontier and the rest score above 0.997, so the BCC model loses its discriminatory power—a well-known small-sample effect, reinforced here by the bounded and homogeneous nature of the output variables across OECD countries. The same pattern emerged under an alternative dataset with mixed reference years, confirming that it is structural rather than data-specific. This empirical result is the principal justification for retaining CRS as the main specification; the VRS and scale-efficiency results are reported for completeness. Since conventional DEA scores are deterministic and ignore sampling variation, the next step corrects for potential bias using the Bootstrap DEA approach.

The Bootstrap results are reported in Table 7, which presents the conventional scores, the bias-corrected estimates, the estimated bias, and the 95% confidence intervals (B=2000). After bias correction, the average score decreases from 0.688 to 0.612, with an average bias of 0.076, confirming that conventional DEA overstates efficiency in this sample. Notably, no country’s confidence interval includes the value of one: once sampling variation is taken into account, no health system in the sample can be regarded as statistically fully efficient.

Mexico and Türkiye deserve particular attention. Both receive a conventional score of 1.000, and both receive by far the largest bias corrections (0.233 and 0.239) with the widest confidence intervals. The reason lies in their input profiles: they have the two lowest levels of health expenditure per capita ($651 and $386, compared with $1219 for the next country) and the two lowest physician densities in the sample. They therefore occupy an extreme region of the input space, where few comparable peer countries exist, and their conventional scores are based on a limited reference set rather than close comparisons with similar countries. The Bootstrap makes this visible: the large bias and wide intervals show that their conventional scores reflect the sparsity of comparable observations as much as genuine performance. Their bias-corrected scores (0.767 and 0.761) still place them in the upper part of the ranking, suggesting that both systems convert very limited resources into reasonable outcomes, though with more uncertainty than the conventional model implies.

The bias-corrected ranking is led by South Korea (0.855), Canada (0.843), and the United States (0.830), none of which are on the conventional frontier. Their position reflects favorable input–output conversion rather than resource levels: South Korea combines moderate health expenditure with the highest life expectancy in the sample, and Canada pairs near-average inputs with strong outcomes. The United States is a more nuanced case: despite the highest health expenditure, it has one of the lowest physician densities, and the weight flexibility of DEA allows its score to rest mainly on the physical-resource dimensions rather than spending. This result should also be read together with the scope of the output set, which does not capture care quality, access, or equity. Since the confidence intervals of these three countries overlap, they should be read as a leading group rather than a strict ranking. At the other end, Greece (0.331), Austria (0.407), and Italy (0.451) form a stable low-efficiency group. Their common feature is not a lack of resources but the opposite: Austria has the highest physician density in the sample, Greece the second highest, and Italy operates with above-average physician numbers. Despite this abundance, their health outcomes remain close to the sample average, so the available capacity is not translated into correspondingly better outputs. Capacity expansion alone, therefore, does not improve measured efficiency; what matters is the conversion of capacity into outcomes. The sources of this conversion gap cannot be identified within the present framework and would require system-level analysis.

The truncated regression model is employed to examine the relationship between the explanatory variables and the bias-corrected efficiency scores. The explanatory variables are GDP per capita, out-of-pocket health expenditure, alcohol consumption, and tobacco use prevalence. The signs of the estimated coefficients show the direction of each relationship, while the Bootstrap confidence intervals indicate the associated uncertainty. Table 8 reports the results with 95% confidence intervals.

All four coefficients are negative. Tobacco use prevalence has a significantly negative association with bias-corrected efficiency ($\hat{\beta }=-0.0120$, 95% CI [−0.0198,−0.0044]): a one percentage point increase in prevalence is associated with a decrease of about 0.012 in the efficiency score. Alcohol consumption is also negative but borderline ($\hat{\beta }=-0.0151$, 95% CI [−0.0333,+0.0038]), with the upper bound slightly above zero. GDP per capita and out-of-pocket expenditure are negative but not statistically significant. These directions are consistent with earlier second-stage evidence for OECD countries, where alcohol and tobacco consumption are associated with lower efficiency scores [9,41,83]. Multicollinearity is not a concern: variance inflation factors range from 1.04 to 2.08.

Overall, the truncated regression shows that behavioral risk factors, particularly tobacco use, are negatively associated with bias-corrected efficiency, whereas the economic variables have a more limited explanatory role. At the same time, a linear specification can only capture average effects and may miss threshold-type patterns, as the borderline result for alcohol suggests. This motivates the machine learning-based analyses that follow. RF and XGBoost are employed to examine the relationships between the bias-corrected efficiency scores and the explanatory variables in a nonlinear setting, and to evaluate potential changes under alternative policy scenarios.

Before turning to the interpretation results, the predictive performance of the two models is reported. LOOCV yields $R^{2}=0.194$ (RMSE =0.122, MAE =0.099) for Random Forest and $R^{2}=0.245$ (RMSE =0.118, MAE =0.099) for XGBoost; repeated 5-fold cross-validation gives results of similar magnitude. These moderate values are expected for a cross-sectional sample of 26 observations. For this reason, the models are used to interpret patterns and explore scenarios rather than to make forecasts, and the partial dependence results below are interpreted only where both models agree. To examine the relationship between bias-corrected efficiency scores and the explanatory variables in more detail, partial dependence analysis is conducted with both the Random Forest and XGBoost models. The results suggest how average efficiency scores may change as a given explanatory variable varies across its observed range, while other variables are held constant. These findings help to identify the ranges in which health system performance appears to be more sensitive to changes in specific variables.

**Figure 2:**
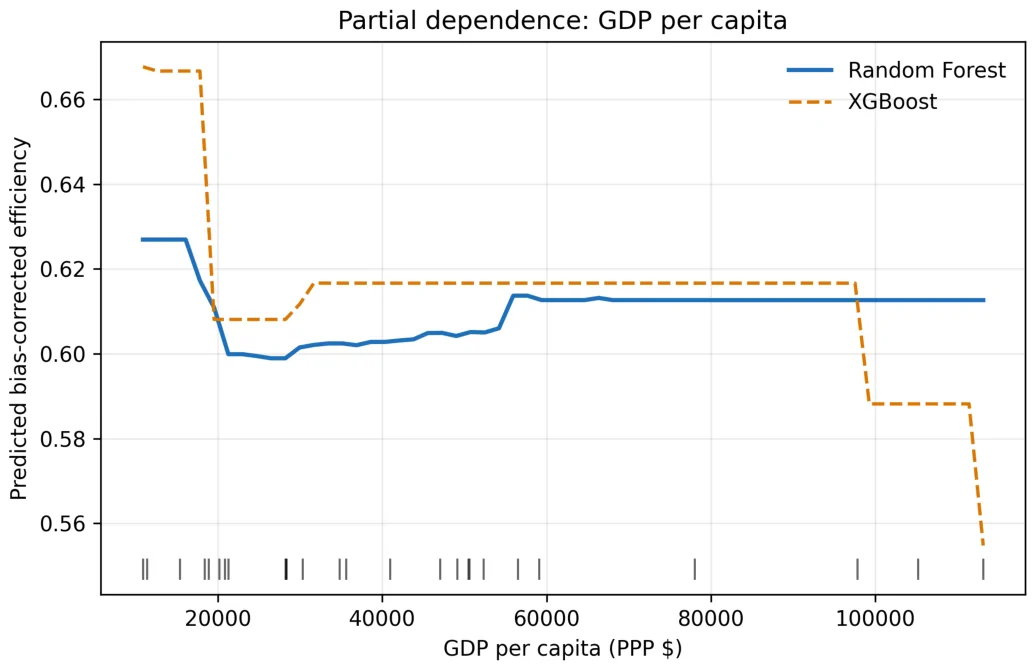
PDA results for GDP per capita.

Figure 2 presents the partial dependence of predicted efficiency on GDP per capita.

Both models show relatively high predicted efficiency at the lowest income levels, a decline up to approximately $20,000–30,000, and an essentially flat profile beyond this range. The two models agree on this overall shape, and the rug marks show sufficient observations across the low and middle income ranges, so the pattern satisfies the interpretation criteria. The flat segment over most of the income distribution indicates that, beyond a moderate income level, additional economic capacity is not associated with higher predicted efficiency. This is consistent with the insignificant GDP coefficient in the truncated regression. The XGBoost curve additionally shows a drop beyond approximately $100,000. However, this segment rests on only two observations (Ireland and Norway); the Random Forest curve remains flat in the same region, and the pattern is therefore not interpreted. Two mechanisms from the literature are consistent with this shape. At low income levels, basic health services deliver the largest health gains at relatively low cost, so widely accessible essential care can produce high efficiency even with limited resources. At higher income levels, in turn, additional spending is increasingly exposed to the well-documented sources of inefficiency in healthcare, namely overuse, underuse, and misuse of services [96,97], which may explain why greater economic capacity does not translate into higher measured efficiency. These mechanisms are offered as possible explanations consistent with the observed pattern rather than as tested findings.

**Figure 3:**
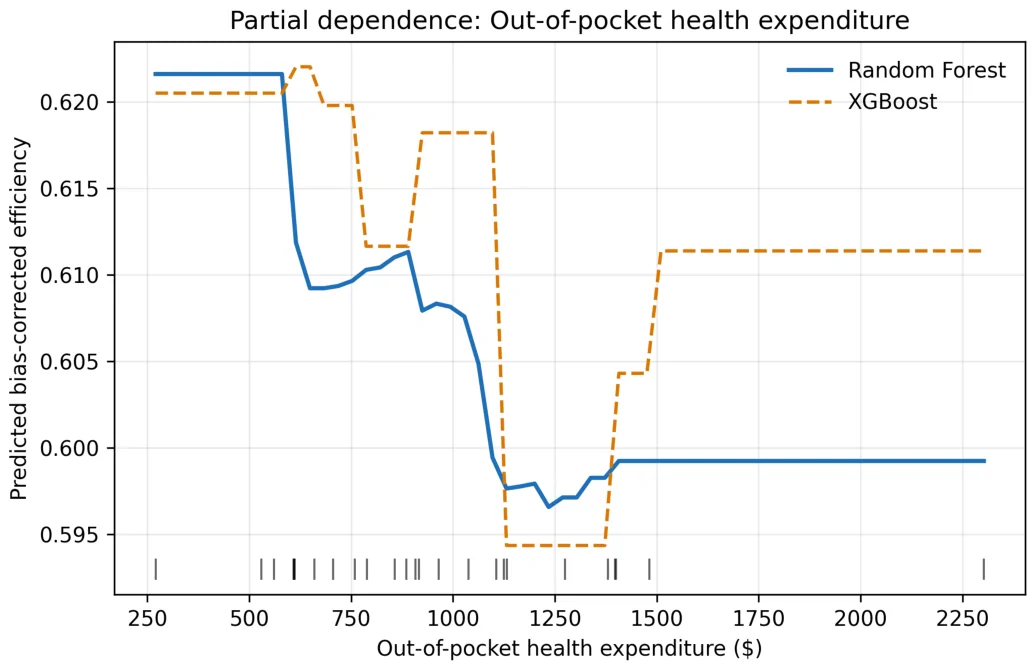
PDA for out-of-pocket health expenditure.

The partial dependence of predicted efficiency on out-of-pocket health expenditure is shown in Figure 3.

Both models show higher predicted efficiency at low expenditure levels, a gradual decline up to approximately $1100 to $1300, and a flat profile beyond this range. The models agree on the overall shape, although XGBoost shows more step-like movements in the middle range. High out-of-pocket payments are commonly associated with limited financial protection and reduced access to care, which can lower service utilization and, in turn, health outcomes. Notably, the three countries with the lowest out-of-pocket levels in the sample (below $600: Türkiye, Mexico, and Poland) all rank in the upper part of the bias-corrected distribution. All three implemented comprehensive health reforms that expanded coverage and strengthened financial protection: the Health Transformation Program in Türkiye (2003–2010) [73,98,99,100,101], the Seguro Popular reform in Mexico (2004–2019) [102,103,104], and the restructuring reforms in Poland (1999–2003) [105,106,107]. Their position may reflect the protective effect of these reforms, although this remains a hypothesis that cannot be tested within the present framework.

**Figure 4:**
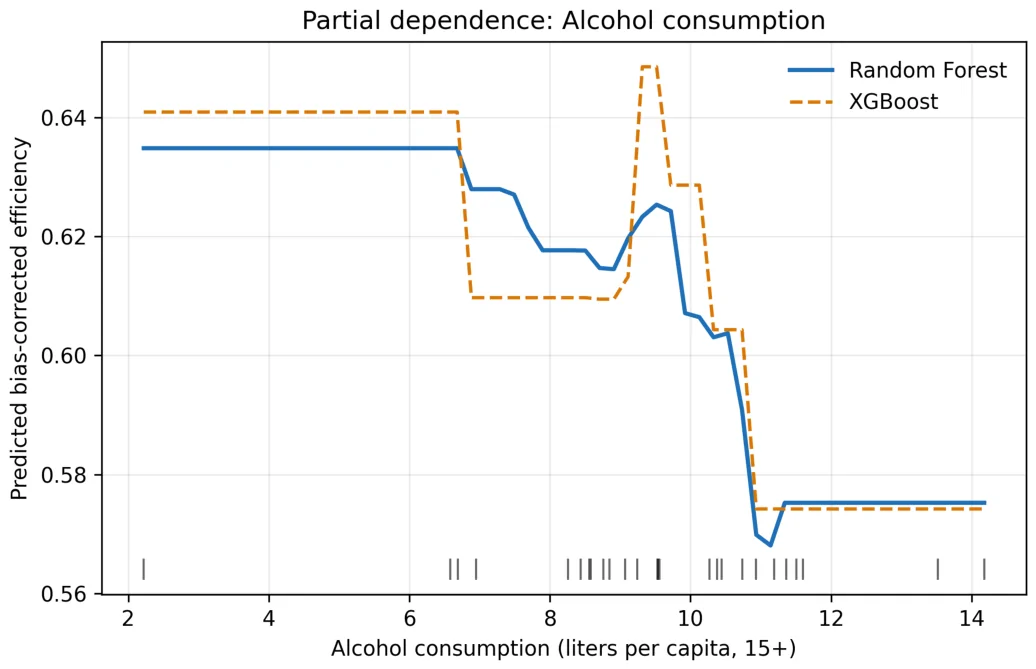
PDA for alcohol consumption.

The partial dependence of predicted efficiency on alcohol consumption is shown in Figure 4.

Both models indicate a decline in predicted efficiency when consumption exceeds approximately 10 to 11 L per capita, and they agree on the direction and location of this drop. This suggests that high alcohol consumption places a burden on health systems that becomes visible beyond a threshold rather than gradually. The flat segment on the left of the curve, however, rests on a single observation (Türkiye, at 2.2 L), with no countries observed between roughly 2 and 6.5 L; this region is therefore not interpreted. The threshold pattern is also consistent with the borderline negative coefficient in the truncated regression: a linear model averages over the flat and declining segments and therefore understates an association that operates mainly at high consumption levels.

**Figure 5:**
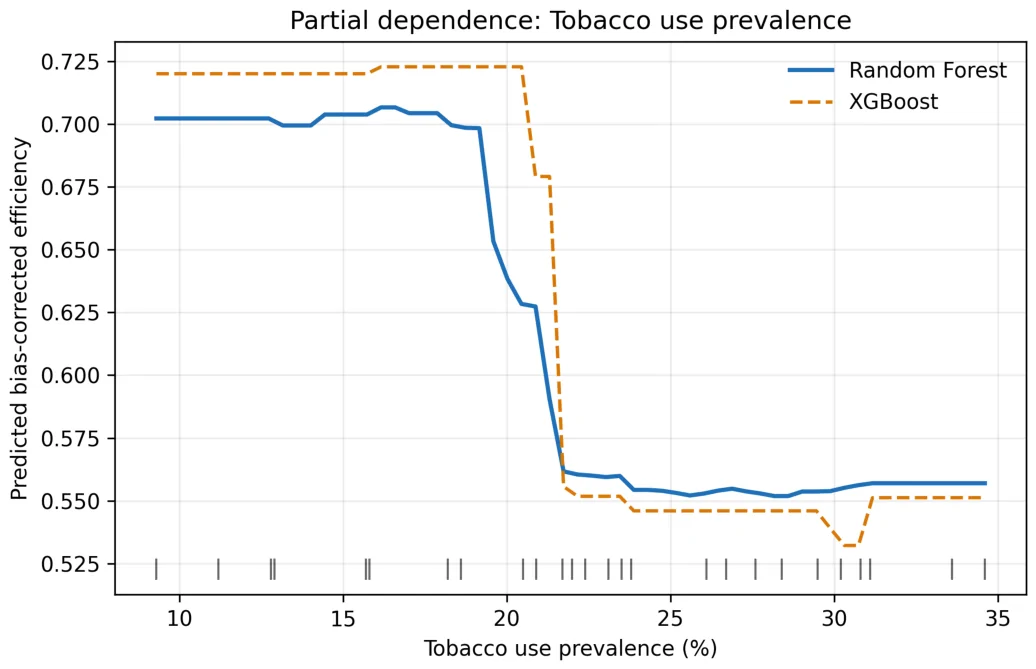
PDA for tobacco use prevalence.

The partial dependence of predicted efficiency on tobacco use prevalence is presented in Figure 5.

This is the strongest and most consistent pattern in the analysis. Both models show stable, high predicted efficiency at low prevalence levels, followed by a sharp decline at approximately 20 to 25 percent prevalence; beyond this range, predicted efficiency settles at a substantially lower level. The two algorithms agree closely on both the location and the magnitude of the drop, and the rug marks show observations on both sides of the threshold, so the pattern satisfies both interpretation criteria. The low prevalence plateau contains only four countries (New Zealand, Canada, the United Kingdom, and Norway), including two of the top bias-corrected performers. This finding is consistent with the significant negative coefficient in the truncated regression and suggests that high tobacco prevalence is associated with a discrete efficiency penalty rather than a gradual decline.

To explore how different policy changes may affect health system performance, a scenario analysis is carried out using machine learning models. Key variables are adjusted according to the intervention levels, and the resulting changes in predicted bias-corrected efficiency scores are examined. The average percentage changes across scenarios, along with model comparisons, are presented in Table 9.

In line with the validation results, these projections should be read as model-based, rather than forecasts.

- Scenario 0 is the baseline with no intervention and serves as the reference for all comparisons.
- Scenario 1, a 10% increase in GDP per capita, produces essentially no change in Random Forest and a small decline in XGBoost. This is consistent with the flat partial dependence profile of GDP and with its insignificant regression coefficient: income growth alone does not translate into predicted efficiency gains.
- Scenario 2, a 10% reduction in out-of-pocket expenditure, yields a small positive change in both models, in line with the modest and gradual pattern observed in the partial dependence analysis.
- Scenario 3, a 10% reduction in alcohol consumption, produces a clear positive change in both models, consistent with the threshold pattern identified above.
- Scenario 4, a 10% reduction in tobacco use prevalence, is the strongest single-factor intervention in both models (+3.56% in Random Forest and +7.23% in XGBoost). This mirrors the sharp threshold in the partial dependence analysis: a 10% reduction moves several countries below the critical prevalence range.
- Scenario 5 combines all four interventions and yields the largest gain in Random Forest (+5.09%) but a slightly smaller gain than the tobacco-only scenario in XGBoost (+6.71% versus +7.23%). The combined effect is therefore sub-additive: the gains from individual interventions overlap rather than accumulate, partly because the negative GDP contribution offsets part of the improvement.

**Figure 6:**
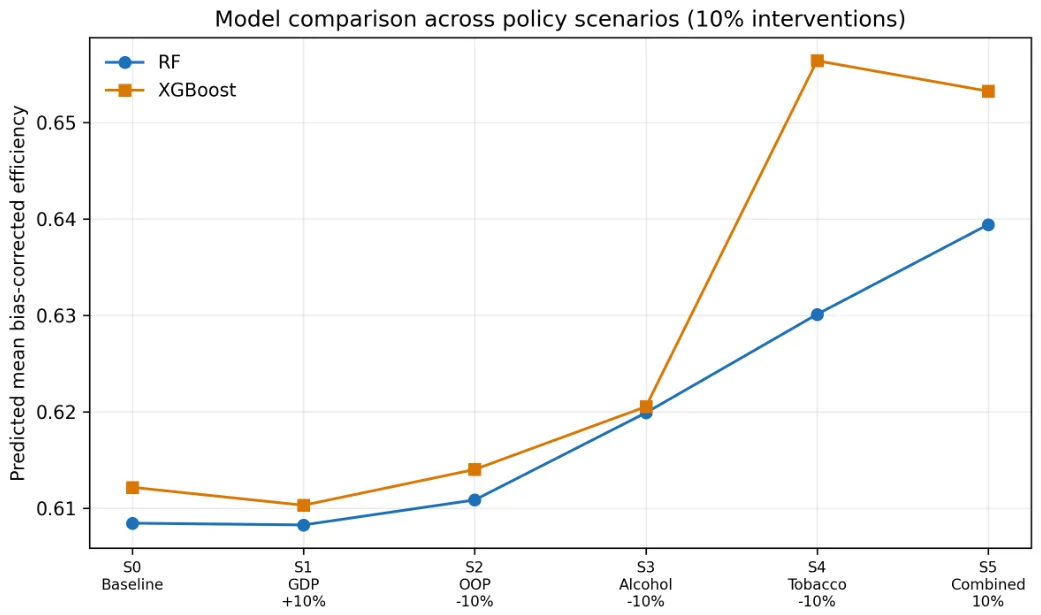
Comparison of predicted mean bias-corrected efficiency scores across scenarios for RF and XGBoost models.

Figure 6 shows how the predicted mean efficiency scores change under the different policy scenarios for both algorithms. The two models follow the same ordering of scenarios, with the tobacco reduction scenario producing the largest single-factor gain in both. XGBoost is more sensitive to changes in the explanatory variables, which is consistent with its steeper partial dependence profiles, while Random Forest gives more stable and conservative predictions. The agreement of the two models on the ranking of interventions, despite their different learning paradigms, supports the robustness of the scenario conclusions.

As a sensitivity check, all scenarios are re-estimated at 5%, 15%, and 20% intervention levels with both models shown in Table 10). Tobacco use reduction remains the strongest single-factor intervention at every level and in both algorithms, with predicted gains rising from +1.80%/+3.51% (RF/XGBoost) at the 5% level to +7.19%/+8.75% at the 20% level. The ordering of the remaining interventions is likewise unchanged across levels, indicating that the scenario conclusions are not sensitive to the specific choice of the 10% magnitude. In XGBoost, the gains grow less than proportionally with the intervention level, which is consistent with the threshold structure identified in the partial dependence analysis: once most countries fall below the critical prevalence range, further reductions yield smaller additional gains.

According to the results of this study, health system performance depends not only on the quantity of available resources but also on how effectively these resources are converted into outcomes and supported by policies. The Two-Stage Bootstrap DEA overcomes the limitations of conventional DEA models, providing more reliable performance measures across OECD countries. The truncated regression shows that tobacco use prevalence is significantly and negatively associated with efficiency, while GDP per capita does not necessarily lead to higher efficiency, indicating that economic growth alone is not sufficient for effective health system functioning. These results highlight the importance of behavioral factors alongside macroeconomic conditions. The scenario analysis shows that policies do not affect predicted performance equally: GDP-based changes show limited or even negative effects, whereas the reduction of tobacco use produces the largest single-intervention gains, and combined interventions add further, yet sub-additive, improvements. This highlights the role of preventive and demand-driven policies, going beyond traditional expenditure-focused approaches. By combining Bootstrap DEA with machine learning-based scenario analysis, the framework helps policymakers assess both current performance and the potential effects of alternative policy options.

The findings suggest that, within the studied sample, higher economic capacity alone is not associated with higher efficiency: GDP per capita is statistically insignificant in the truncated regression, and a simulated 10% GDP increase yields no predicted efficiency gain in either machine learning model. In contrast, behavioral risk factors show consistent negative associations. Tobacco use prevalence is significantly negative in the truncated regression, both algorithms identify a sharp efficiency decline at approximately 20–25% tobacco prevalence; a simulated 10% tobacco reduction produces the largest single-intervention improvement, robust across intervention levels of 5–20%; and the Supplementary SHAP analysis assigns tobacco the largest variable importance in both models. These convergent results across four independent analytical approaches support prioritizing preventive and financial-protection policies over expenditure expansion alone, although the cross-sectional design precludes causal claims.

## 5. Conclusions and Future Work

This study presents a data-driven framework that combines Two-Stage Bootstrap DEA with machine learning-based scenario analysis to assess health system performance. Developed using data from 26 OECD countries, this approach contributes to the health system performance literature by providing bias-corrected technical efficiency scores together with the potential impacts of alternative policy interventions. The findings suggest that higher economic capacity alone is not associated with higher efficiency, whereas interventions targeting behavioral risk factors—tobacco use, in particular—are associated with the largest predicted improvements. In terms of policy, the scenario analysis points to a clear priority ordering: a 10% reduction in tobacco use prevalence yields the largest predicted single-intervention gain in both models (+3.6% and +7.2%), followed by alcohol reduction, while reducing out-of-pocket payments adds a smaller improvement and GDP growth alone adds none. These results suggest that, within this sample, tobacco control is the single most promising lever for efficiency improvement, and that combined packages should be designed around it rather than around expenditure expansion.

This study has some limitations. First, the analysis covers 26 countries. This is sufficient for the DEA stage, where the Bootstrap corrects the small-sample bias, but it limits the machine learning stage: predictive accuracy is moderate (LOOCV $R^{2}$ between 0.19 and 0.25), which is expected for a sample of this size. The models are therefore used to interpret patterns and explore scenarios, not to make forecasts. Second, the outputs (life expectancy and infant survival) capture the main health outcomes but not care quality, access, or equity; the efficiency scores show how well countries convert resources into health outcomes, not the overall quality of their systems. Third, the data are cross-sectional, so the results show associations rather than causal effects. Finally, the findings apply to the OECD context and may not hold for very different health systems.

Methodologically, the proposed framework offers a flexible tool for policy assessment by combining nonparametric efficiency analysis with machine learning techniques. Future studies could address these limitations and improve this approach by including broader country groups, adding additional variables such as demographic and digitalization indicators, and using panel data structures to better capture changes over time. Although this study relies on cross-sectional data, the growing availability of real-time data from interconnected health information systems presents promising opportunities for future research. Integrating DEA with continuously updated data streams, such as electronic health records, digital surveillance systems, and other health information platforms, could enable dynamic monitoring of health system performance and provide more timely evidence to support policy responses during public health emergencies. In parallel, improving the transparency and interpretability of machine learning models through explainable AI techniques will be important for ensuring that such data-driven policy assessments remain understandable and actionable for decision-makers [108]. This study demonstrates that combining robust efficiency measurement with scenario-based analysis can provide valuable insights for both researchers and policymakers in increasingly complex health systems. More importantly, it suggests that lasting improvements in efficiency depend not only on the level of resources but also on how those resources are used and supported by appropriate policies.

## Figures and Tables

**Table 1:** Comparison of the proposed framework with selected studies.

Study	Context	Bias Correction	Second Stage	ML	ML Validation	Scenario Analysis
*Bootstrap DEA Studies in Health Systems*						
Marschall and Flessa [32]	Burkina Faso, PHC facilities	✓	Trunc. + Tobit	–	–	–
De et al. [33]	Indian states	✓	Tobit	–	–	–
Kim and Kang [34]	170 countries	✓	–	–	–	–
See and Yen [37]	121 countries	✓	Trunc. reg.	–	–	–
Asghar et al. [38]	49 OIC countries	✓	–	–	–	–
Andrews [39]	New Zealand DHBs	✓	Beta reg.	–	–	–
Trakakis et al. [40]	Greek health centers	✓	Trunc. reg.	–	–	–
Konca and Top [41]	36 OECD countries	–	Tobit	–	–	–
Zulfakhar Zubir et al. [43]	Malaysia, 30 years	✓	Trunc. reg.	–	–	–
*DEA–Machine Learning Hybrid Studies in Health Systems*						
An et al. [45]	Online health platform, China	–	–	✓	–	–
Chuang et al. [46]	Taiwanese hospitals	–	–	✓	–	–
Cosgun et al. [47]	US states, vaccination	–	–	✓	✓	–
Cosgun et al. [48]	US nursing homes	–	–	✓	✓	–
Sun et al. [49]	US counties, telehealth	–	–	✓	✓	–
**Originality**	**26 OECD health systems**	✓	**Trunc. reg. + Bootstrap CI**	✓	✓	✓

**Table 2:** Variables used in the DEA framework.

Variable	Definition	Year	Data Source	References
**Inputs**				
Health expenditure	Health expenditure per capita measured in purchasing power parity (PPP, $).	2022	WHO	[51,61,62]
Number of physicians	Number of medical doctors per 10,000 population.	2022	WHO	[2,9,51,63,64,65]
Number of hospital beds	Number of total hospital beds per 10,000 population.	2022	WHO	[9,51,61,63,65]
**Outputs**				
Life expectancy	Life expectancy at birth.	2022	WHO	[25,30,51,64,66,67,68,69,70]
Infant survival rate	1−(infantmortalityrate/1000)	2022	WHO	[27,67,71,72,73]
**Explanatory Variables**				
GDP per capita	Gross domestic product per capita measured in purchasing power parity (PPP).	2022	OWID	[74,75,76]
Out-of-pocket health expenditure	Per capita health expenditure paid directly by households and not covered by public insurance.	2022	OWID	[77,78,79]
Alcohol consumption	Total alcohol consumption per capita among individuals aged 15 years and older.	2022	WHO	[52,75,80,81,82]
Tobacco consumption	Estimated prevalence of current tobacco use (%, age-standardized).	2022	WHO	[52,65,75,80,81,83]

**Table 3:** Descriptive statistics of the study variables.

Variable	Minimum	Maximum	Mean
**Inputs**			
Health expenditure	385.90	12,586.00	4230.14
Number of physicians	22.36	65.76	38.06
Number of hospital beds	10.14	128.10	44.62
**Outputs**			
Life expectancy	74.50	83.50	80.18
Infant survival rate	0.9889	0.9983	0.9962
**Explanatory Variables**			
GDP per capita	10,897.84	113,122.10	43,216.25
Out-of-pocket health expenditure	270.07	2302.44	956.98
Alcohol consumption	2.22	14.18	9.50
Tobacco consumption	9.30	34.60	22.73

**Table 4:** Policy scenarios.

Scenario	Scenario Description
S0	Current data are used, and no policy intervention is applied.
S1	A 10% increase in gross domestic product (GDP) per capita.
S2	A 10% reduction in out-of-pocket health expenditures.
S3	A 10% reduction in alcohol consumption.
S4	A 10% reduction in tobacco use prevalence.
S5	All four interventions (S1–S4) implemented jointly.

**Table 5:** Model summary information.

Model Characteristic	Specification
Model type	Data Envelopment Analysis
Number of DMUs	26 OECD countries
Number of inputs	3
Number of outputs	2
Explanatory variables	4
Orientation	Output-oriented
Returns to scale	CRS
Bootstrap method	Simar–Wilson, Two-Stage procedure
Bootstrap replications (*B*)	2000
Confidence intervals	95%
Bias correction	2× original score − mean Bootstrap score
Second-stage model	Truncated regression
Machine learning models	Random Forest and XGBoost
Model validation	LOOCV
Random seed	42
Software	R (version 4.4.2) and Python (version 3.12.13).

**Table 6:** Technical efficiency and scale efficiency scores.

Country	TE (CRS)	TE (VRS)	Scale Efficiency
Mexico	1.000	1.000	1.000
Türkiye	1.000	1.000	1.000
South Korea	0.917	1.000	0.917
United States	0.909	1.000	0.909
Canada	0.901	1.000	0.901
Chile	0.828	1.000	0.828
United Kingdom	0.805	1.000	0.805
Ireland	0.768	1.000	0.768
Belgium	0.722	1.000	0.722
Slovenia	0.713	1.000	0.713
New Zealand	0.705	0.999	0.705
Netherlands	0.689	1.000	0.689
Latvia	0.662	1.000	0.662
Poland	0.656	1.000	0.656
Hungary	0.650	1.000	0.650
Spain	0.625	1.000	0.625
Slovakia	0.607	0.998	0.608
France	0.604	0.999	0.605
Switzerland	0.574	1.000	0.574
Norway	0.564	1.000	0.564
Estonia	0.564	1.000	0.564
Czechia	0.541	1.000	0.542
Italy	0.518	1.000	0.518
Germany	0.514	0.999	0.514
Austria	0.442	0.999	0.442
Greece	0.402	1.000	0.402
**Average**	**0.688**	**0.999**	**0.688**

**Table 7:** Bootstrap DEA results: Bias-corrected efficiency scores with 95% confidence intervals (B=2000).

Country	Efficiency	Bias-Corrected	Bias	95% CI
	Score	Score		
South Korea	0.917	0.855	0.061	[0.781, 0.910]
Canada	0.901	0.843	0.058	[0.773, 0.892]
United States	0.909	0.830	0.079	[0.770, 0.888]
Mexico	1.000	0.767	0.233	[0.649, 0.977]
Türkiye	1.000	0.761	0.239	[0.642, 0.982]
United Kingdom	0.805	0.744	0.060	[0.668, 0.795]
Chile	0.828	0.719	0.108	[0.626, 0.811]
Ireland	0.768	0.719	0.049	[0.657, 0.761]
Belgium	0.722	0.673	0.048	[0.616, 0.718]
New Zealand	0.705	0.657	0.048	[0.603, 0.699]
Slovenia	0.713	0.650	0.063	[0.593, 0.702]
Netherlands	0.689	0.626	0.064	[0.547, 0.683]
Latvia	0.662	0.590	0.072	[0.522, 0.654]
Poland	0.656	0.571	0.085	[0.499, 0.649]
Hungary	0.650	0.570	0.080	[0.496, 0.645]
Spain	0.625	0.563	0.062	[0.492, 0.619]
France	0.604	0.558	0.047	[0.511, 0.598]
Switzerland	0.574	0.539	0.035	[0.496, 0.568]
Slovakia	0.607	0.537	0.070	[0.475, 0.600]
Estonia	0.564	0.499	0.064	[0.443, 0.553]
Norway	0.564	0.489	0.075	[0.424, 0.557]
Czechia	0.541	0.488	0.054	[0.436, 0.536]
Germany	0.514	0.477	0.037	[0.436, 0.510]
Italy	0.518	0.451	0.066	[0.391, 0.510]
Austria	0.442	0.407	0.035	[0.375, 0.437]
Greece	0.402	0.331	0.071	[0.285, 0.394]
**Average**	**0.688**	**0.612**	**0.076**	—

**Table 8:** Truncated regression results with parametric Bootstrap 95% confidence intervals (B=2000).

Variable	$\hat{\beta }$	Std. Error	Lower CI (2.5%)	Upper CI (97.5%)
Intercept	1.1150	0.1569	0.8035	1.4286
GDP per capita	−0.0007	0.0011	−0.0030	0.0016
Out-of-pocket expenditure	−0.0058	0.0067	−0.0189	0.0080
Alcohol consumption	−0.0151	0.0097	−0.0333	0.0038
Tobacco use prevalence	−0.0120	0.0039	−0.0198	−0.0044

**Table 9:** Scenario analysis: Average percentage change in predicted bias-corrected efficiency relative to baseline (10% intervention level).

Scenario	Random Forest (Δ%)	XGBoost (Δ%)
S0: Baseline	0.000	0.000
S1: GDP per capita (+10%)	−0.030	−0.304
S2: OOP expenditure (−10%)	0.396	0.302
S3: Alcohol consumption (−10%)	1.882	1.366
S4: Tobacco use prevalence (−10%)	3.560	7.227
S5: Combined scenario	5.090	6.712

**Table 10:** Sensitivity of the strongest single-factor intervention across intervention levels.

Level	Strongest Single-Factor Intervention	RF (Δ%)	XGBoost (Δ%)
5%	Tobacco use reduction	+1.80	+3.51
10%	Tobacco use reduction	+3.56	+7.23
15%	Tobacco use reduction	+5.23	+7.92
20%	Tobacco use reduction	+7.19	+8.75

## Data Availability

The data used in this study are available from the authors upon reasonable request.

## Supplementary Materials

The following supporting information can be downloaded at https://www.mdpi.com/article/10.3390/healthcare14162536/s1, S1: SHAP summary plot.

## Abbreviations

The following abbreviations are used in this manuscript:

CCR	Charnes, Cooper, and Rhodes
CRS	Constant Returns to Scale
DEA	Data Envelopment Analysis
DMU	Decision-Making Unit
GDP	Gross Domestic Product
ML	Machine Learning
OECD	Organisation for Economic Co-operation and Development
OWID	Our World in Data
PDA	Partial Dependence Analysis
PPP	Purchasing Power Parity
RF	Random Forest
XGBoost	Extreme Gradient Boosting
WHO	World Health Organization
