# Supplementary material for: Beyond Efficiency Scores: Explaining Health System Performance Using Two-Stage Bootstrap DEA and Machine Learning
Source: Healthcare (Basel). 2026 Aug 13;14(16):2536. doi: 10.3390/healthcare14162536 (PMC13512731; doi:10.3390/healthcare14162536)
Supplement: Supplementary file 1 [file healthcare-14-02536-s001.zip › healthcare-4441977-supplementary.pdf]

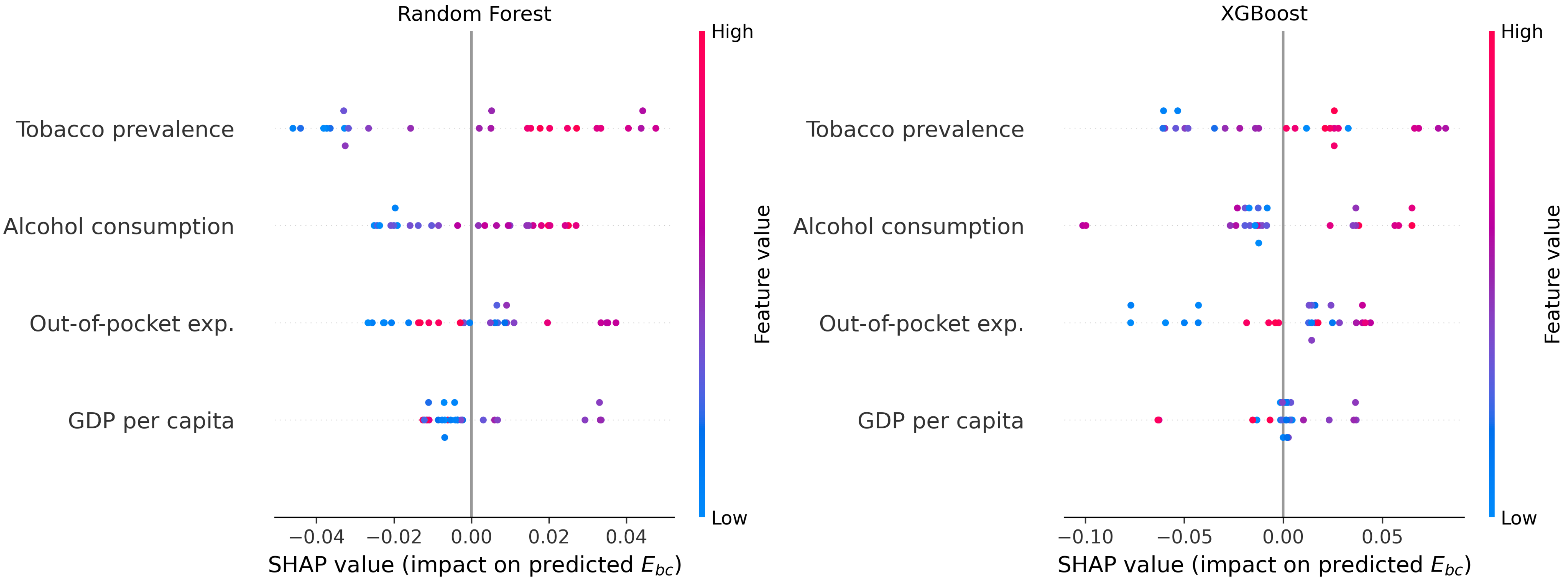
Random Forest
XGBoost
Tobacco prevalence
Alcohol consumption
Out-of-pocket exp.
GDP per capita
High
Low
Feature value
−0.04
−0.02
0.00
0.02
0.04
−0.10
−0.05
0.05
SHAP value (impact on predicted E_{bc})
